# Supplementary figures and images for: Motivations of potential anchor businesses to support community development and community health
Source: PLoS One. 2022 Jul 27;17(7):e0269400. doi: 10.1371/journal.pone.0269400 (PMC9328504; doi:10.1371/journal.pone.0269400)

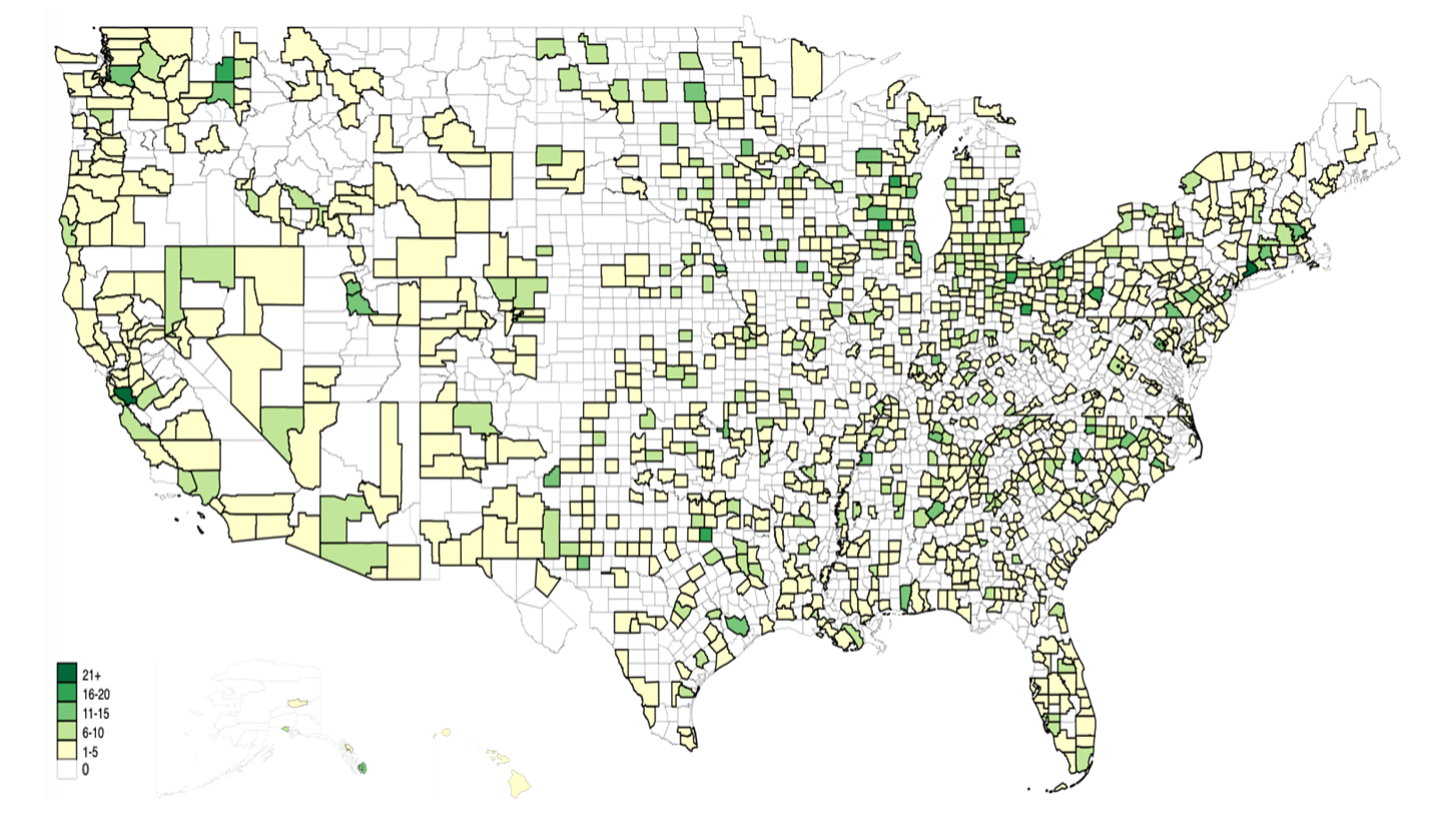

Supplement: S1 Fig — (TIF) [file pone.0269400.s001.tif]
