# Supplementary material for: Motivations of potential anchor businesses to support community development and community health
Source: PLoS One. 2022 Jul 27;17(7):e0269400. doi: 10.1371/journal.pone.0269400 (PMC9328504; doi:10.1371/journal.pone.0269400)
Supplement: S1 File — (PDF) [file pone.0269400.s002.pdf]

Variable	Description
headquarters location	City, State for company headquarters
zip code	Zipe code for company headquarters
county	County of company heaquarters
date	Date
URL	Company website URL
family owned?	Is this company “family owned”?
co-op?	Is this company a"CO-OP"? (i.e. community owned)
mission statement?	Is the mission statement available?
mission text	Relevant text (copy and paste)
mission justice/equity	Mention of creating a more democratic, just and equitable society or community
mission local focus	Mention of its business’ focus on a specific geographic area or community
mission CSR	Corporate social responsibility
mission community development	Community development or community investment
mission sustainability or environmental	Sustainability or environmental impact
mission health or wellness	Health or wellness (beyond worker on the job safety)
mission none of the above	None of the above mentioned in the mission statement or other stated goals of the company
mission other	Other?
local supplies	Does the company mention in its mission statement or on its website or in its annual report an intention to purchase from or other commitment to purchase materials from local suppliers?
hire local	Does the company mention in its mission statement or on its website or in its annual report an intention to hire local residents?
local business support	Does the company mention in its mission statement or on its website or in its annual report an intention to form and assist local businesses?
sustainable/ethical materials	Does the company mention in its mission statement or on its website or in its annual report that it purchases sustainable or ethnically-sourced materials from vetted suppliers?
2019 revenue	2019 revenue in millions of USD

chamber of commerce	Is the company part of a local chamber of commerce or another business group that would indicate community influence?
other	ex. won awards for giving and local involvement
