# Supplementary material for: Motivations of potential anchor businesses to support community development and community health
Source: PLoS One. 2022 Jul 27;17(7):e0269400. doi: 10.1371/journal.pone.0269400 (PMC9328504; doi:10.1371/journal.pone.0269400)
Supplement: S2 File — (PDF) [file pone.0269400.s003.pdf]

Background on Study

Thank you for agreeing to talk with us today.

[Introduce self and others who are on the call]

Let me start by briefly giving you background on the study we are conducting.

The Robert Wood Johnson Foundation has funded the RAND Corporation, a nonprofit and nonpartisan independent research organization based in the United States, to conduct a study to determine why (or why not) some for-profit companies choose to invest in community health or other community development.

In the following questions, we will refer to [COMPANY NAME] as “your company”.

When I refer to the “local community” or “surrounding community”, I mean the town, city or county in which your company is located.

When I refer to “social responsibility” initiatives I am referring to beneficial physical or social changes that result in community growth, stability or revitalization. This could be focused on economic development, such as training the local workforce or directing procurement to local vendors. Or this could be community development, which often is charitable in nature, such as building parks, supporting affordable housing, investing in public education or donating to local food banks.

In this conversation, we are interested in learning what your company does, its role within the local community, and why the company has or had not made social responsibility initiatives and investments in local community development.

I would like to mention a few procedural points around privacy and confidentiality:

- Your participation is voluntary and you can stop at any time for any reason.
- Your responses to our questions as well as your participation will not be reported to anyone outside this RAND project team, including others at your company.
- You may decline to discuss (that is, skip) any topic that we raise.
- While your identity, organization, and individual answers will not be disclosed beyond our research team, we will reference characteristics of your organization (e.g., industry, employment size categories) in the report resulting from the study.
- We will be reporting themes and variation in responses across the interviews. We may include some direct quotes, but will not be attributing them to anyone by name or position in a way that could directly identify you. However, some people who know this field may make inferences, correctly or not, about the source of the quotes.

Do you have any questions? (YES/NO)
Do you agree to participate? (YES/NO)

With your permission, we would like to record this conversation. This helps us ensure we've accurately captured our conversation. Once we have our notes in order, we will destroy the audio-recording.

- **Do I have your permission to record our conversation?** (YES/NO)
- [If respondent grants permission, begin recording]
    - [Note to interviewer: Pause here to quickly provide some identifying information for recording purposes]
        - Interview Date
        - Interviewer Name
        - Respondent Name, Title, and Organization

Interviewee Role

I would like to begin by making sure I understand your role at your company.

1. What is your current title?
    - Can you please tell me about your position with regard to interaction and support of with the local community?
2. How long have you been working for your company? How long have you been working in this position?

About Your Company

3. What are your company's key products or services?
4. What towns, cities, or areas are most important to your company's operations? (e.g., company headquarters, important plants or offices)?
   [Note: answer to this question will determine the communities we will discuss below.]
   For how long has your company's U.S. headquarters been in its current location?

Economic Ties

In the following questions, we refer to […towns, cities, areas interviewee mentioned above] as the surrounding communities.

5. *Consumers*:
    a. Who is your primary customer?
    b. Are your core customers primarily from the surrounding communities?
6. *Suppliers*:
    c. What type of suppliers are needed for operations at your company's core locations?
    d. Are they primarily located in the surrounding communities?
7. *Community influence*:
    e. Is your company among the top 10 employers in terms of the influence on the surrounding communities? If not, is it among the top 20, 50, or 100?

f. In 2019, would you say your company was a key driver of economic activity in the surrounding community? That is, if your company moved to another location, what would happen to the surrounding community economically?

8. *Immobility*:
   g. What percent of your company's assets are invested in fixed assets, such as real estate, or infrastructure, in this community?
   h. How difficult would it be financially or otherwise for your company to relocate to a new community on a scale of 1 to 10, 1 being least difficult, 10 being most difficult? Why?

About The Community In Which Your Company is Rooted

9. In your opinion, does the surrounding community have considerable needs for development?
   (e.g., poverty, poor infrastructure, natural disaster, poor population health, etc.)
   a. If no, was there a need in the past 5 years? Please explain.
   b. If yes, please explain.

Company Community Investments

10. Please describe your company's beliefs about corporate social responsibility and a business' role in community development, if any?
    a. For example, is social responsibility – either economic or community development - part of your company's mission, business strategy, and/or ethical standards?
    b. If so, what's the goal of your corporate social responsibility policy?
    c. Does your company consider developing surrounding communities and ensuring equity as part of its responsibility, such as improving housing, education, health, and safety?

11. Has your company made social responsibility investments that support the local community in the past 5 years, such as training the local workforce outside employees, building local infrastructure, offering affordable housing, and improving community health and public safety? If yes,
    a. Please describe these investments [Note to interviewer: verify the location of each investment initiative]
    b. Were these investments health related?
    c. For what % of company revenue (or profits) do these investments account?

12. Has your company initiated programs that improve surrounding communities through business operations such as directing purchases to local suppliers, or prioritizing hiring local residents?

Investment Rationale

13. [IF INVESTMENT] You mentioned that your company has made improvements in surrounding communities

    i. What are the motivations? [Note to interviewers: prompt if needed, improve customer base, strengthen workforce, goodwill etc.]
    j. Who made the decisions?
    k. What is the decision process? What factors facilitated those decisions?
    l. Are these initiatives operated by a philanthropy arm (e.g., a separate department, or a foundation) or part of your core business operation?

14. It seems that your company has invested in initiatives directly related to health such as [examples from discussion]:
    a. Is there a specific reason your company invested in these programs directly related to health? (Ask about specific initiatives discovered via the environmental scan or mentioned in this conversation)

15. It seems that your company has invested in social responsibility initiatives not directly related to health as [examples from discussion]:
    a. Are there a specific reasons your company invested in these [non health-related] specific areas? (Ask about specific initiatives discovered via the environmental scan or mentioned in this conversation)

16. [IF NO INVESTMENT] Did your company ever consider initiating programs to improve surrounding communities? If yes, please describe what investments or initiatives were considered but not initiated.

    [IF YES]
    b. Please describe the decision-making process and how your company arrived at the current decision.
        i. [IF HEALTH RELATED INITATIVES CONSIDERED] Why did your company decide not to invest in programs directly related to health?
        ii. [IF NON-HEALTH RELATED INITIATIVES CONSIDERED] Why did your company decide not to invest in these [non health-related] specific areas?

[ASK ALL]

17. What are the barriers to new or additional company investments in community development initiatives?
18. How have or could these be resolved?
19. Are there any differences in terms of existing barriers to investing in health-related vs. non-health related community initiatives?
    c. If so, how could these be resolved differently?

Concluding Questions

20. Is there anything else you would like to share about the topics we have just discussed?
21. Is there anything else you feel I should be asking about this topic when speaking to company representatives, such as yourself?

Thank you again for participating. I am now going to turn off the recording [turn off recording]
