# Supplementary material for: Motivations of potential anchor businesses to support community development and community health
Source: PLoS One. 2022 Jul 27;17(7):e0269400. doi: 10.1371/journal.pone.0269400 (PMC9328504; doi:10.1371/journal.pone.0269400)
Supplement: Attachment — Submitted filename: PONE-D-22-02864_reviewer.docx [file pone.0269400.s005.docx]

**Title:** Motivations of anchor businesses to support community development and community health

**Reviewer Comment 1:** I think you have strong results and conclusions; however, I think your background lacks a bit of context about the need for anchor institutions in America. Federal and State retrenchment from health, education, local economies, housing, poverty, addressing social ills/SDOH, etc. led to more localized and commercial initiatives to address such. We have no national health system for example. Essentially, neoliberal political economics paved the way for the rise and need for privatized anchors. I think this could be resolved in one brief sentence, or part of a sentence, simply mentioning the growing need due to Federal and State institutions removing themselves from such responsibilities, leaving the onus on private/commercial institutions. I think you did well explaining motivations to anchor in your introduction/background.

**Reviewer Comment 2**: Local hiring and supplier contracts, and support for local businesses are integral to community health, particularly the social determinants of health [lines 235-237]. How do the low rates here reflect on the broader definition of anchor institutions as businesses that embed themselves in the community and contribute to the health [social/economic] of the community? I am wondering if your data provided you with any information about why these rates are so low? Is ownership type a factor in why these rates are lower? I understand if you do not have the data to respond to this comment, but it is a primary question I have after reviewing the entire manuscript and thinking about your larger conclusions about for-profit anchors. Could for-profit anchors being anchoring in different ways due to different organizations/capital structures and constraints (including being tax paying entities)? Perhaps they are more focused on acute economic developments as opposed to systemic? Or – is this related to your qualitative data where some participants mentioned they do not understand why larger health initiatives are not a priority? If so, how can such initiatives be leveraged? What are the perceived barriers to such initiatives (this is generally addressed in section 3.5)? Line 487-489 somewhat addresses my questions, could this section be a bit more contextual about what initiatives are included in “community development” and what that means for our understanding and classification of for-profits as anchors? I do see this is addressed as a limitation [lines 562-563 = health v non-health generalizations]; however, any clarification or context would be appreciated.

**Reviewer Comment 3:** Overall, I think this research contributes to the literature on understanding corporate anchors in the US.
