# Supplementary material for: Motivations of potential anchor businesses to support community development and community health
Source: PLoS One. 2022 Jul 27;17(7):e0269400. doi: 10.1371/journal.pone.0269400 (PMC9328504; doi:10.1371/journal.pone.0269400)
Supplement: Attachment — Submitted filename: Response to Reviewers.docx [file pone.0269400.s006.docx]

**Response to Reviewer Comments**: Motivations of potential anchor businesses to support community development and community health

**Reviewer #1:**

| **Comment** | **Response** |
| --- | --- |
| I think you have strong results and conclusions; however, I think your background lacks a bit of context about the need for anchor institutions in America. Federal and State retrenchment from health, education, local economies, housing, poverty, addressing social ills/SDOH, etc. led to more localized and commercial initiatives to address such. We have no national health system for example. Essentially, neoliberal political economics paved the way for the rise and need for privatized anchors. I think this could be resolved in one brief sentence, or part of a sentence, simply mentioning the growing need due to Federal and State institutions removing themselves from such responsibilities, leaving the onus on private/commercial institutions. | Thank you for your comment. We have updated the introduction to point to these additional factors, including a new citation.  “In the absence of a national health system and the ongoing retrenchment of the Federal and State governments [3], however, many local organizations are taking new innovative approaches to uplift affected communities.” lines 47-50 |
| I think you did well explaining motivations to anchor in your introduction/background. | Thank you. We appreciate your comment. |
| Local hiring and supplier contracts, and support for local businesses are integral to community health, particularly the social determinants of health [lines 235-237]. How do the low rates here reflect on the broader definition of anchor institutions as businesses that embed themselves in the community and contribute to the health [social/economic] of the community? I am wondering if your data provided you with any information about why these rates are so low? Is ownership type a factor in why these rates are lower? I understand if you do not have the data to respond to this comment, but it is a primary question I have after reviewing the entire manuscript and thinking about your larger conclusions about for-profit anchors. | This is an excellent point. It’s possible that these connections or policies are not reported publicly and thus not captured in our research. However, these companies represent more diverse business operations than “eds and Meds”. Based on some qualitative interviews where the supply chain was discussed, interviewees stated that the supplies needed for business operations weren’t usually available for purchase through the community (specifically, financial management software platforms, electricity and specialized farm equipment). However, we do not feel that we have achieved data saturation on this point to include this information in our paper. Thus we have added to the discussion section:  ”Further, few companies in our sample had policies to source from local suppliers, which could mean that these policies are not public and were not captured in our environmental scan, or unlike “eds and meds”, supplies for these for-profit business operations aren’t available locally. Future work is needed to determine if the type of business operations influences if potential anchor businesses choose local suppliers and contracts.” lines 731-735 |
| Could for-profit anchors being anchoring in different ways due to different organizations/capital structures and constraints (including being tax paying entities)? Perhaps they are more focused on acute economic developments as opposed to systemic? Or – is this related to your qualitative data where some participants mentioned they do not understand why larger health initiatives are not a priority? If so, how can such initiatives be leveraged? What are the perceived barriers to such initiatives (this is generally addressed in section 3.5)? Line 487-489 somewhat addresses my questions, could this section be a bit more contextual about what initiatives are included in “community development” and what that means for our understanding and classification of for-profits as anchors? I do see this is addressed as a limitation [lines 562-563 = health v non-health generalizations]; however, any clarification or context would be appreciated. | The reviewer asks very interesting questions, which we are unfortunately unprepared to fully answer based on our existing work that primarily focuses on motives.  It is conceivable that for-profit anchors behave differently compared to non-profit anchors because for-profits have to pay taxes and need to earn a market-level return on investment. Even within for-profits, capital-intensive companies (e.g., real estate or some large manufacturers) may have different priorities than those with less-intensive investments in a local area. Prior literature suggests that involvement in local community development is also associated with being a consumer-oriented business; they might want to reach out to local consumers and create a reputable brand. With regard to health vs. non-health priorities, our data and analysis yielded some helpful insights as described in the text. For example, some company representatives believe that investments in health take a long time to see results that may or may not turn out to be what is expected.  Of course, more research is needed to understand these issues better.  As the reviewer mentioned, the limitations section addresses this specifically - though in response to this comment, we add the reviewer’s point about capital structures and tax implications:  “As for-profit entities, there may be tax incentives or capital structures in place that favor some types of community development over others.” lines 777-779 |

**Reviewer #2:**

| **Comment** | **Response** |
| --- | --- |
| The authors provide a nice summary of the literature in terms of definitions of anchors and anchor businesses. | Thank you. We appreciate your comment. |
| More effort needs to focus on the motivation for this study. They correctly note that much is known about drivers of business contributions to community development by for profit businesses, but less is known about anchor businesses. Why would the motivations of business anchors be different than general for-profit businesses, which also include business anchors? And there have been many representatives from anchor businesses who have discussed their organizations’ motivations for community contributions at conferences on this topic (National Academy of Medicine, RWJF Forums, US Chamber of Commerce Forums). | According to our knowledge, there is published literature exploring the motivations for non-profit anchor institutions, but little research specifically on for-profit anchor institutions (anchor businesses). In addition, the studies on factors (or characteristics) associated with contributions among for-profit businesses did not investigate motivation.  “However, most literature on this topic is broadly about the characteristics of such for-profit businesses, whereas the motivations behind anchor businesses and potential anchor businesses to contribute to community development is less understood.” lines 106-108 |
| Methods. The methods section needs more detail. For example, what information does DonorSearch contain regarding nonpolitical and non-foundation contributions? The $50K threshold strikes me as very low, particularly when companies often point to their foundations as being the main source of community contributions, and those contributions can be millions of dollars for large businesses. For example, a $50K annual contribution from a large distribution warehouse employing potentially hundreds or thousands of workers strikes me as unsubstantial and not a meaningful investment in the community. | The reviewer has a point that $50k for a large company is not a meaningful contribution. There are two possible reasons. First, the amount did not include contributions from company foundations or political donations. Second, there seems to be a large variation in contribution size.  In the paper, we note that the $50K threshold was empirically-defined (section 2.3) and that many potential anchor businesses did donate more during that time period (see Table 2 describing the environmental scan sample and Table 3 describing the interview sample).  With regard to whether this cut-off is meaningful, we devote a paragraph of our limitations section to discussion of this threshold. (beginning “A limitation of our work was that we determined what was considered to be a threshold for substantial investment,...” beginning on line 767  Further, we have added additional details on the information collected from DonorSearch.  “The DonorSearch database collects information about which organizations donate to charitable causes or organizations and the amount they donate as a part of their philanthropic efforts. Data is categorized by types of donations (i.e., political donations, annual donations, in kind donations, etc.) with information on the source of the data (typically, the organization’s annual report).” lines 195-199 |
| I’m wondering why the team included areas with > 1 million residents as businesses are less likely to have a local or reginal influence in these areas. Particularly if they are only contributing $50K. | In this study, we were interested in identifying potential anchor businesses in the U.S. and did not want to limit areas of the U.S. based on assumptions about population density. Thus, we included all MSAs in the US.  We believe what matters is the size of a company relative to the community. Therefore, we applied the revenue and employment ratio thresholds (i.e., measures relative to the local MSA) to identify potential anchor businesses. And these thresholds were derived from exemplar anchor businesses. Those businesses meeting the thresholds are “potential” anchor businesses, which may or may not contribute to community development or contribute a relatively small amount.  The study was designed to investigate among “potential” anchor businesses why some contributed while others did not. Excluding areas with more than one million residents could generate a biased sample for our purpose.  Further, we used the empirically-defined $50K threshold, admittedly a conservative threshold of what might be considered “substantial”, so that we had consistent requirements across the US. We also note that the $50K threshold was a minimum and that many potential anchor businesses did donate more during that time period (see Table 2 and Table 3). |
| I suspect think there’s a big difference in motivation toward community contribution between companies that are one of several within a community and those that are the only large business in the community. | A good point. We found very few communities with multiple potential anchor businesses (see Supplementary Figure), which again, is defined relatively to the revenue and employment in the headquarters’ surrounding MSA. The interviews we conducted were of organizations in areas with one identified potential anchor business, so we do not have the information needed to compare potential anchor businesses in areas with other local businesses. However, some company representatives did express that they avoided the areas that others have already invested in. Also, according to the unpublished studies sponsored by the Robert Wood Johnson Foundation, it seems for-profits are more likely to work with non-profits than other for-profits, and there is a lack of coordination among for-profits. |
| “To confirm economic ties to the community, we collected information regarding whether the company hired local, sourced their supplies locally, supported local business development or participated in their local chamber of commerce.” Where did the authors collect this information from? I cannot imagine that this is available on websites beyond a vague/broad description. And what were the thresholds used? | Each of these 4 variables the reviewer lists was binary (yes/no), thus no threshold determination or judgment call needed. To clarify, we have added additional detail to the methods to document our process for identifying these economic ties.  “We conducted internet searches and reviewed each company’s US website, online publications, and annual reports for the above information for our systematic environmental scan, including public companies’ filings to the Security Exchange Commission, where applicable” beginning on line 315  We have also submitted our abstraction tool as an appendix (S2) for further clarity. |
| Some of the information in the Methods section is vague. What data specifically came from SEC filings? What were the 25th percentiles for employment and revenue? It would be helpful if the team shared the data collection tool and protocol in an appendix. | We have clarified that the SEC filings were used to fill in information not otherwise available on the website in the environmental scan. In addition to the changes described above, we clarified the employment and revenue ratios that we used:  “We determined those anchor businesses’ respective economic footprint in their headquarters’ community differed from matched pairs in the proportions of employment and revenue for which the company was responsible within the company headquarters’ MSA, i.e., the employment ratio - the number of company employees at the headquarters to total working population of the headquarter’s MSA, and the revenue ratio - that ratio of total company revenue to total revenue in MSA. As the goal was to include a vast majority of potential anchor businesses, we considered them to be a potential anchor business if a business met a threshold that was either the 25th percentile of the example anchor businesses' employment ratio or the revenue ratio (0.016% and 0.034%, respectively).” lines 149-158  As per the reviewer’s request, we now share the data collection tool and protocol as an appendix. |
| Section 2.3, I would caution the authors against using the term “representative sample,” regarding the interviews. | We have removed the mention of “representative sample” from the text and clarified our sampling procedure.  “To ensure we would interview comparable businesses across the 3 categories outlined above, we started our selection of companies to recruit by randomly selecting 4 businesses from a stratified sample from group 1.” line 285-301 |
| Can the authors clarify whether they only interviewed one person per company? In looking at the interview protocol, I’m surprised that a single person could give correct answers to all of these questions. For example, “What percent of your company’s assets are invested in fixed assets, such as real estate, or infrastructure, in this community?” and “Please describe your company’s beliefs about corporate social responsibility and a business’ role in community development, if any?” | We have clarified in the text that we interviewed one or more persons per company. The companies decided which individuals and roles to include based on the description of our study provided in our recruiting emails.  “We conducted semi-structured interviews over Microsoft Teams video conference following a discussion guide intended to steer a 30-60 min conversation with one or more participants per company (See S1 File).” lines 317-319 |
| Also, how would the authors handle a company respondent who said that they were not in the top 10 employers in terms of influence on the surrounding communities (and therefore not meeting the anchor business definition)? Was there any basis for the respondents assessments? | We asked for respondent’s self-reported beliefs about being a top 10 employer in terms of influence on the surrounding community. We did not use this as a criterion to screen business. If the respondent did not agree they were, we would probe on their response to understand why they did not think so considering that we had selected these companies based on relative revenue and employment. In alignment with our sampling strategy, we did not encounter a case where a respondent did not think their employer was a top 10 employer in terms on influence on their surrounding community. |
| Looking through the results, I’m realizing that there may be a discrepancy between the giving profile of a company, and that of a branch/plant in a particular community. This is an issue for a large company headquarted in on area, but with branches/plants/warehouses in other areas. Are the dollars described in the manuscript specific to a specific community, or is that the total dollar value across the US (or internationally)? Is this study looking at headquarters only? | For simplicity, our study focused on headquarters locations of companies. The giving / contributions are specific to the MSA where the headquarters is located. We have identified this in the text:  “We focused on contributions to the community surrounding their US headquarters, excluding political donations and those made from company foundations, as contributions from these foundations may represent funds obtained from other entities in addition to the company.” line 167-170 |
| Some respondents were from philanthropy, but in the Methods section, philanthropy dollars were not included in the analysis. | We have added text to the draft to clarify why people from philanthropy were included while dollars from the non-profit entities affiliated with these companies were not used.  “Individuals from philanthropy departments were included because they oversee the giving activities of the company, even though philanthropy dollars were not included in assessments of anchor businesses due to the ability for philanthropy dollars to include company funds as well as funds from other individuals or organizations.” lines 313-316 |
| Only 8 of 57 respondents agreed to participate? This strikes me as particularly problematic. | We agree that the response rate was not ideal and addressed this in our limitations section: “Our sample is more representative than prior case studies, or studies using convenience samples or targeting the largest companies or particular industries. Nonetheless, our sample was subject to non-response bias. Having recruited for interviews during the COVID pandemic, potential interviewees were working remotely and thus may have been more difficult to reach. Companies had closed, merged, and been purchased, and may have experienced staff turnover. Further, the positions of interest to this study may have been terminated, leading to higher non-response. Compared to respondents, non-respondents were larger companies in terms of revenue but contributed much less to community development. As a result, non-response bias limits the generalizability of our findings.” lines 750-758  Further, in response to this comment, we have added a note to Table 3 and revised our supplementary figure to give more details about the companies we attempted to contact but did not participate. Among these were entities we determined to be ineligible (i.e., out of business, mislabeled non-profit), declined to participate, did not respond and had no person or persons at the company who could answer our questions. |
| I thought the study was focused on for-profit anchor institutions, but some of the results appear to include “Eds and Meds”. | We include only for-profit companies in our study (i.e., “Given the resources available to for-profit institutions and their great potential to contribute to surrounding communities, it is important to understand which for-profit institutions can be considered as potential anchor businesses, where they are located, and why they make contributions to community well-being.” line 109-112 )  Unfortunately, we do not understand what prompted this comment and thus are unable to address it. |
| Overall, I’m a bit underwhelmed by the findings and have concerns about the methods and large number of non-participants. | We hope that our responses above will help alleviate the reviewer’s concerns around methods and we acknowledge the limitations of the study. That said, we believe that our paper adds needed foundational work describing potential anchor businesses across the U.S., especially as no consensus definition exists for these companies, as well as those businesses’ philosophies and reasons for contributing to their local communities. |

**Reviewer #3:**

| Comment | Response |
| --- | --- |
| Overall, I think this research contributes to the literature on understanding corporate anchors in the US. | Thank you for your comment. |
| Clarity in details throughout the paper would help strengthen it further. Specifically, I would encourage the authors to include more context about the data sets used. | Thank you for your input. We have added additional details about the databases used.  “The Dun & Bradstreet database collects and organizes data on key metrics of businesses in the US, including their revenues, number of employees, and organizational structure (i.e., whether a company is a subsidiary, its for-profit status, etc.).”  “DonorSearch database collects information about which organizations donate to charitable causes or organizations and the amount they donate as a part of their philanthropic efforts” |
| Additionally, more context regarding the rationale for specifically including and identifying health-related initiatives would be helpful. The authors may also find recent work by Choyke/Cronin/Franz helpful in discussing health-related anchors. Please see additional, more specific points of feedback below: | We include both health and non-health related initiatives in our analysis (health and non-health contributions). Our focus was on health-related initiatives to understand how for-profits think of and invest in health, drawing comparisons to those for-profits who invest in primarily non-health activities. We have included a citation by the Choyke/Cronin/Franz group where we briefly discuss similarities in our findings. |
| L79-80 - Worth noting this quality isn't unique to for-profits; nonprofits often benefit similarly. | We have updated the text to note this is also true for non-profits.  “There is a general consensus in the literature that for-profit and non-profit businesses’ contributions to community development generate indirect benefits to the companies themselves through improved reputation, employee pride, or local business environment…” |
| L120-121 - Recommend clarifying why organizations are considered anchors (e.g., large employer, economic impact, etc.). | We have clarified why these organizations are considered anchors in the text. Please see section 2.1. Identifying Potential Anchor Businesses. |
| L143 - One example of a database that should be explained in more detail (purpose, etc.). | We have added some additional information about the Dun & Bradstreet database for context.  “The Dun & Bradstreet database collects and organizes data on key metrics of businesses in the US, including their revenues, number of employees, and organizational structure (i.e., whether a company is a subsidiary, its for-profit status, etc.).” lines 136-142 |
| L164 - More information regarding the DonorSearch database would be helpful as well. | We have added some additional information about the DonorSearch database for context.  “DonorSearch database collects information about which organizations donate to charitable causes or organizations and the amount they donate as a part of their philanthropic efforts” lines 195-199 |
| L238-239 - The importance of the environmental sustainability policies could use clarification. | We have added some additional information about why environmental sustainability is important.  “…and 13% of companies had specific policies around environmental sustainability as a part of their Environment, Social, and Corporate Governance (ESG) goals.” lines 355-356 |
| L243-244 - Examples would be helpful in understanding the nature of health references in mission statements. | We have added some examples to the text to clarify what is meant by health references in mission statements.  “…29% had a specific focus on health in their mission statements (such as improving access to care or insurance),” lines 360-362 |
| L420-L425 - This paragraph is confusing - more clarification is needed to understand why health would not be a strength of a health contributor. | Thank you for pointing out that that paragraph was confusing. We have revised it to clarify the concepts expressed by the interviewees:  “When potential anchor business representatives discussed how health-focused and non-health focused community development differs, the most common concept described was a desire to fit within an ecosystem of local charitable giving (health contributor #1, non-health contributor #1 and health contributor #4). Within a geographic area, companies wanted to “coordinate resources in communities to make sure that all the investment is done effectively” (health contributor #4). That is, they wanted to avoid overlapping or competing with other companies’ investments in the same community and further “to successfully leverage each other” (health contributor #4). That said, these potential anchor businesses, including one health contributor had a perception that health initiatives should be the responsibility of other companies for which these initiatives would be more aligned with a business ‘strength’.” line 564-573 |
